# Severe Hepatopulmonary Syndrome in a Child with Caroli Syndrome

**DOI:** 10.1155/2017/2171974

**Published:** 2017-08-13

**Authors:** W. De Jesus-Rojas, K. McBeth, A. Yadav, J. M. Stark, R. A. Mosquera, C. Jon

**Affiliations:** Department of Pediatrics, Division of Pulmonary Medicine, McGovern Medical School at University of Texas Health Science Center, Houston, TX, USA

## Abstract

Hepatopulmonary Syndrome (HPS) is a potential complication of chronic liver disease and is more commonly seen in the adult population. Caroli Syndrome is a rare inherited disorder characterized by intrahepatic ductal dilation and liver fibrosis that leads to portal hypertension. In children with liver disease, HPS should be considered in the differential diagnosis of prolonged, otherwise unexplained, hypoxemia. The presence of HPS can improve patient priority on the liver transplantation wait list, despite their Pediatric End-Stage Liver Disease (PELD) score. We present a 6-year-old girl with Caroli Syndrome and End-Stage Renal Disease who presented with persistent hypoxemia. The goal of this report is to increase awareness of HPS in children.

## 1. Background

The triad of arterial hypoxemia, chronic liver disease, and evidence of intrapulmonary vascular dilation define Hepatopulmonary Syndrome (HPS) [1, 2]. Fluckiger described the first patient with symptoms of HPS in 1884, but Knudson first characterized HPS as a disease in 1977 [3]. Since then, the diagnostic criteria for HPS have been modified (Table 1) to include oxygen impairment, evidence of pulmonary vascular dilation, liver disease, and portal hypertension [4]. The degree of HPS severity is graded by the partial pressure of oxygen (PaO_2_) on an arterial blood gas and the Alveolar-arterial (A-a) gradient. In pediatrics, the prevalence of HPS has been documented as 4–29% in children with cirrhosis or severe portal hypertension, although this may be an underestimation [5]. HPS is a progressive disease that is unlikely to resolve without liver transplantation [6]. Aside from supplemental oxygen, liver transplant is the only treatment option for HPS patients [7]. In adults, the 5-year survival rate improves drastically after liver transplantation [8]. To the best of our knowledge, no data has been published on survival rates in posttransplant children. Patients with Caroli Syndrome are at a high risk of developing portal hypertension and liver failure due to recurrent cholangitis and biliary obstruction. An association between Caroli Syndrome and HPS has not been established.

## 2. Case Presentation

Patient is a 6-year-old girl with Caroli Syndrome, autosomal recessive polycystic kidney disease, and end-stage renal disease (ESRD) status postbilateral nephrectomy on hemodialysis. In addition, she has chronic liver disease with portal hypertension. She was born by C-section at 30 weeks' gestation to a 37-year-old G4P2A1 mother with pregnancy complicated by late prenatal care—last 6 weeks of pregnancy. Due to subglottic stenosis secondary to chronic mechanical ventilation and dysphagia, she required tracheostomy tube placement and gastrostomy, respectively. At baseline, she was on room air via capped tracheal cannula during the day and tracheostomy collar while asleep. Patient presented to the emergency department with 2-week history of hypoxemia even after resolution of a viral illness that required brief hospitalization. Patient required oxygen via a Heat Moisture Exchanger (HME) with T-oxygen connector at 1-2 LPM continuously. She had increased tracheal secretions, which grew* E. coli* ESBL that was treated with 10 days of tobramycin and 7 days of meropenem based on sensitivities. Nephrology Department was consulted and ruled out volume overload as her cause of oxygen requirements. Pulmonary Department was consulted to evaluate additional sources of hypoxemia.

On physical examination, patient was noted to be awake, alert, and in no apparent distress. Respiratory examination revealed symmetric air entry, with no signs of increased work of breathing. There were transmitted central airway rhonchi throughout but no wheezes or crackles. Abdominal exam revealed distension, no tenderness, normoactive bowel sounds, hepatomegaly, and splenomegaly.

**Figure 1:**
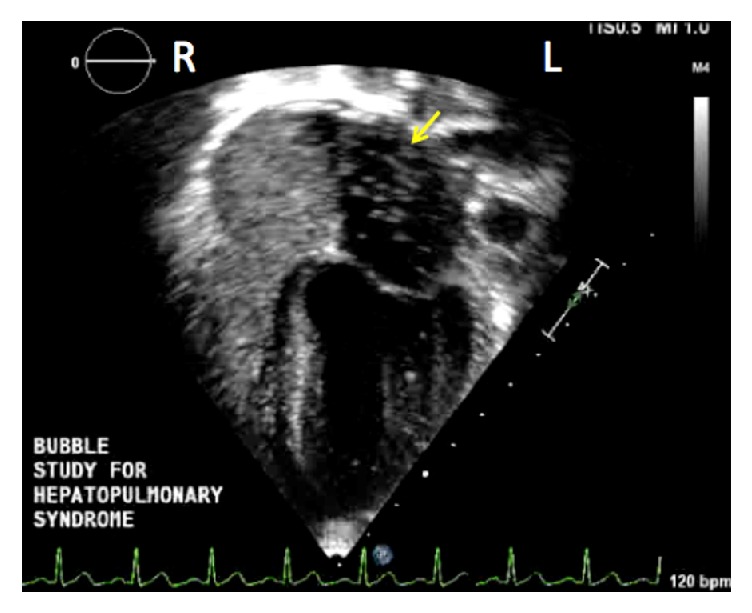
Two-dimensional contrast echocardiography showed the presence of bubbles in the left atrium after 7 cardiac cycles (yellow arrow), consistent with an intrapulmonary shunting (R: right, L: left).

Laboratory results were as follows: WBC: 7.6, Hgb: 9.7, Hct: 29.7, and platelets: 184 with a differential of neutrophiles: 42.7, monocytes: 11.8, lymphocytes: 44.3, and eosinophils: 1.0. Metabolic panel was normal. AST was 20 units/L, ALT was 16 units/L. CRP level was <2.9. HBsAg was negative. Prealbumin was 28.5 mg/dL. PT was 15 s, PTT was 31.8 s, and INR was 1.16. Chest X-ray showed stable cardiomegaly with decreased pulmonary vascular congestion and no pulmonary edema. Due to hepatomegaly, an abdominal ultrasound elastography was performed that showed moderate-to-severe stiffness of the liver with a moderate amount of ascites in the right and left lower quadrants. An abdominal CT scan showed mild biliary ductal dilatation and ascites associated with portal hypertension/hepatic cirrhosis, which is consistent with her history of Caroli Syndrome. An arterial blood gas on room air showed pH: 7.43, PCO_2_: 50, PO_2_: 51, HCO_3_: 33, BE: 8, and Sat: 87% with a calculated A-a gradient of 36 mmHg (normal < 15). A two-dimensional bubble-contrast echocardiography revealed the presence of bubbles in the left atrium at seven cardiac cycles after the right atrium, which is consistent with pulmonary recirculation (Figure 1). No intracardiac shunts were detected. A contrast bubble-echocardiogram, performed 2 months previously, showed normal findings. Technetium-99 macroaggregate albumin study was not obtained.

## 3. Outcomes and Follow-Up

Patient was discharged with supplemental oxygen as needed by tracheostomy collar. Given the diagnosis of HPS, her status was prioritized on the liver-kidney transplantation waitlist.

## 4. Discussion

HPS is the result of a progressive vasodilation of pulmonary vessels secondary to chronic liver failure. No previous case reports have described an association between Caroli Syndrome and HPS. The pathogenesis of HPS is not well understood. Vasodilator mediators, such as nitric oxide (NO) and endothelin-1, have been implicated in the etiology of HPS [9, 10]. However, irrespective of the pathogenic process, it is known that vasodilatation of the pulmonary capillaries occurs.

The hypoxemia associated with HPS is related to ventilation-perfusion mismatch and oxygen diffusion limitation. Increased blood flow across enlarged pulmonary vessels and capillaries impair adequate saturation of hemoglobin due to right to left shunt. Concurrently, the distance that oxygen travels across the alveolar-capillary membrane is increased which impedes oxygen's accessibility to hemoglobin binding sites and thus results in hypoxemia. In contrast to an intracardiac shunt defect, oxygen supplementation corrects the hypoxemia due to V/Q mismatch and diffusion defect that are seen in HPS patients. True anatomic shunts between arteries and veins can occur in HPS but are rare [11].

Diagnostic criteria of HPS include evidence of chronic liver disease, impaired oxygenation, and evidence of intrapulmonary shunting. Evidences of PaO_2_ of <80 mmHg with an elevated A-a gradient (>15 mmHg) are required [7] diagnostic criteria. Presence of intrapulmonary shunting with a two-dimensional contrast echocardiography or a Technetium-99 macroaggregate albumin nuclear test should be documented. A positive echocardiography demonstrates presence of contrast in the left side of the heart after three cardiac cycles from the right atrium. More than 6% brain uptake of Technetium-99 should be documented to consider a positive test [7].

Congenital hepatic fibrosis is one of the fibropolycystic diseases, which also includes Caroli disease. The pathophysiology of Caroli Syndrome involves the presence of a progressive liver fibrosis and intrahepatic ductal dilatation that predisposes patients to portal hypertension, ascites, and biliary lithiasis [12]. Though rare, patients with pure congenital hepatic fibrosis may also develop complications related to portal hypertension, including varices bleeding and ascites. We speculate that the associated hepatic fibrosis seen in Caroli's Syndrome in this patient predisposed to the development of portal hypertension that, in turn, promoted the development of ascites [13].

No direct relationship has been described between Hepatopulmonary Syndrome and Caroli Syndrome. We speculate that progressive liver damage with portal hypertension as sequelae of a congenital liver disorder may have similar implications to chronic liver failure in adults. Screening for HPS in patients with Caroli Syndrome should be considered in patients with unexplained hypoxemia.

Our patient showed a triad of hypoxemia and chronic liver failure with portal hypertension secondary to Caroli Syndrome and evidence of a contrast echocardiography consistent with intrapulmonary shunting. Contrast echocardiography is preferred over the Technetium-99 macroaggregate albumin in the detection of intrapulmonary shunting [14]. Due to her history of chronic liver failure and end-stage renal disease she was placed on the liver-kidney transplant list. The PELD score estimates severity of the liver disease and approximates the likely survival for patients who are waiting for a transplant. HPS is considered an exception diagnosis for the PELD score and provides additional points to raise the patient's position on the liver transplant waitlist [15]. Awareness about HPS in the pediatric patients with chronic liver failure is an important factor to consider in the multidisciplinary management of HPS in order to expedite liver transplantation and decrease mortality in this population.

## Figures and Tables

**Table 1:** Hepatopulmonary Syndrome diagnostic criteria.

Variable	Criterion
Oxygenation impairment	Partial pressure of oxygen < 80 mmHg or Alveolar-arterial oxygen gradient ≥ 15 on room air

Pulmonary vascular dilation	Presence of contrast in the left side of the heart after three cardiac cycles on a contrast echocardiography OR abnormal uptake in the brain (>6%) with a Technetium-99 macroaggregate albumin study

Liver disease	Portal hypertension with or without cirrhosis

Level of severity	A-a oxygen ≥ 15 plus
Mild	A partial pressure of oxygen ≥ 80 mmHg
Moderate	A partial pressure of oxygen ≥ 60 mmHg to <80 mmHg
Severe	A partial pressure of oxygen ≥ 50 mmHg to <60 mmHg
Very severe	A partial pressure of oxygen < 50 mmHg

Adapted from [7].

## Additional Points

*Learning Points.* (i) The triad of arterial hypoxemia, chronic liver disease, and evidence of intrapulmonary vascular dilation define Hepatopulmonary Syndrome (HPS). (ii) HPS is the result of a progressive vasodilation of pulmonary vessels secondary to chronic liver failure. (iii) Any patient with chronic liver disease, including Caroli Syndrome, should be screened for HPS. (iv) Differential diagnosis of hypoxemia should always include intrapulmonary shunts. (v) Awareness about HPS in the pediatric population may increase patients' PELD scores that can improve the likelihood that they will be transplanted and improve patient survival.
